# Can Genetics Predict Sports Injury? The Association of the Genes *GDF5*, *AMPD1*, *COL5A1* and *IGF2* on Soccer Player Injury Occurrence

**DOI:** 10.3390/sports6010021

**Published:** 2018-03-05

**Authors:** Kiah McCabe, Christopher Collins

**Affiliations:** Muhdo Health Ltd., Ipswich IP4 2BN, UK; info@muhdo.com

**Keywords:** genetics, injury, sport, soccer, DNA, inflammation, football

## Abstract

Genetics plays an integral role in athletic performance and is increasingly becoming recognised as an important risk factor for injury. Ankle and knee injuries are the most common injuries sustained by soccer players. Often these injuries result in players missing training and matches, which can incur significant costs to clubs. This study aimed to identify genotypes associated with ankle and knee injuries in soccer players and how these impacted the number of matches played. 289 soccer players, including 46 professional, 98 semi-professional and 145 amateur players, were genetically tested. Ankle and knee injuries and the number of matches played were recorded during the 2014/15 season. Four genes were assessed in relation to injury. Genotypes found to be associated with injury included the TT (nucleobase) genotype of the *GDF5* gene, TT and CT (nucleobase) genotypes of *AMPD1* gene, TT genotype of *COL5A1* and GG (nucleobase) genotype of *IGF2* gene. These genes were also associated with a decrease in the number of matches played.

## 1. Introduction

Soccer is one of the most commonly played sports in the world, with The Fédération Internationale de Football Association (FIFA) estimating that 265 million people play the sport worldwide, as of 2006. Injury of the lower extremities accounts for approximately two thirds [1] of all injuries among soccer players, with ankle injuries being reported as to be the most common injury. While knee injuries, such as anterior cruciate ligament rupture, causes the most time lost from competition and training in soccer [1]. Injury can have a number of consequences, both to the injured player and to the club. An elite player who is frequently injured during his career may struggle to achieve maximum skill level due to missing training and matches. Injury to a player can have an impact on the club in terms of the number of potential competitive matches missed and the cost incurred. Some research suggests that an injury can lead to 4 missed matches on average [2].

In order to prevent soccer-related injuries, the main risk factors for injury must be identified. Recently, genetic predisposition to injury has become a popular area of research. A number of genes that may play a role in injury have been identified, namely rs143383 (*GDF5*), rs17602729 (*AMPD1*), rs12722 (*COL5A1*) and rs680 (*IGF2*). The *GDF5* gene encodes the protein Growth differentiation factor 5 which is known to influence both the growth and maintenance of bones, muscles and tendons. There are three variants of this gene rs143383, CC, CT and TT. Those expressing a T allele produce less GDF5 protein, which has been shown to be associated with conditions such as osteoarthritis [3,4,5]. The *AMPD1* gene encodes the enzyme Adenosine monophosphate deaminase 1, which is found in skeletal muscle. There are three variants of this gene, CC, CT and TT. Those with a CC genotype have a normal outcome with no deficiency in the enzyme and those with a T allele have a deficiency in the AMPD1 enzyme. Deficiency in AMPD1 can cause symptoms such as pain, cramping and weakness of muscles after exercise [6,7]. The *COL5A1* gene encodes the protein Collagen alpha-1(V) chain, which is involved in the formation of connective tissue in the musculoskeletal system. People with a T allele of this gene have been shown to have an increased risk of injury [8]. The *IGF2* gene encodes insulin-like growth factor 2. This gene is thought to be involved in exercise induced muscle damage [9].

The aim of this study was to identify which genes and genotypes are associated with the most common injuries (ankle and knee) in soccer players and to determine the impact this has on the number of matches played. Identification of these genes may lead to an inexpensive tool that coaches can use to better personalise a soccer player’s training and nutrition to prevent injury from occurring.

## 2. Methods

### 2.1. Participants 

The study involved 289 male soccer players (aged 18–32) including 46 professional, 98 semi-professional and 145 amateur soccer players. The number of separate ankle and knee injuries and number of matches played were recorded during the 14/15 season. Exact injury type was unspecified (including impact versus repetitive and contact versus non-contact). Total injuries calculated as ankle injuries + knee injuries. Each player will have had different training loads based upon their level, motivation and injury/illness profile. Of the 46 professional players 22 were 18 years old, 11 were 21 years old, 5 were 19 years old and 8 were 24 years old. Of the 98 semi-professional players 32 were 18 years old, 23 were 24 years old, 19 were 25 years old, 5 were 29 years old, and 19 were 30 years old. Of the 145 amateur players 35 were 18 years old, 11 were 20 years old, 3 were 21 years old, 42 were 25 years old, 8 were 26 years old, 20 were 28 years old, 5 were 30 years old and 21 were 32 years old.

### 2.2. Sample Collection

289 Samples were collected at the end of the 14/15 season, one for each player, these were collected over an 8–15 week period, the injury data was already gathered by team medical departments, coaches at the end of the season. Samples were taken on iso-helix buccal swabs SK-1S and tested by an iso9001 certified lab. DNA data need only be gathered once in a person’s lifetime due to the rigid nature of the genes analysed and the rarity of mutation (Collins, 2017). Due to this fact, the timing of the swab samples and injury are of little importance; epigenetic data which may be changeable was not analysed in this study.

### 2.3. Data Analysis

Data was analysed using the SPSS (v.24) software package (IBM, UK). The average differences for each genotype were evaluated using one-way ANOVA and post-hoc Bonferroni was applied for multiple comparisons between groups. Correlation between matches played and injury was assessed using Pearson’s correlation, using a two-tailed analysis. Data was considered statistically significant when the P value was less than 0.05.

## 3. Results

### 3.1. GDF5 Genotype Effect on Injury and Matches Played

**Figure 1:**
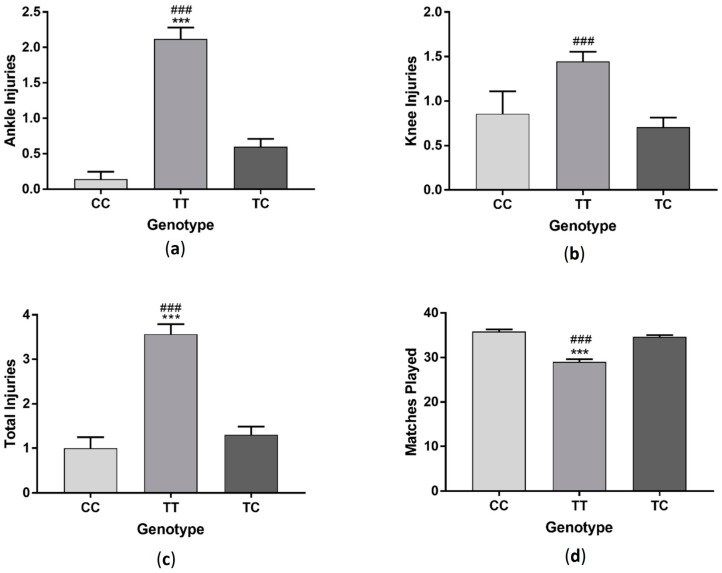
Effect of *GDF5* (rs143383) genotype on ankle injuries (**a**); knee injuries (**b**); total injuries (**c**) and matches played (**d**) in a group of soccer players. *** *p* < 0.001 compared to CC genotype, ^###^ *p* < 0.001 compared to TC genotype. Data shown as Mean ± S.E.M. (Standard error of the mean).

**Figure 2:**
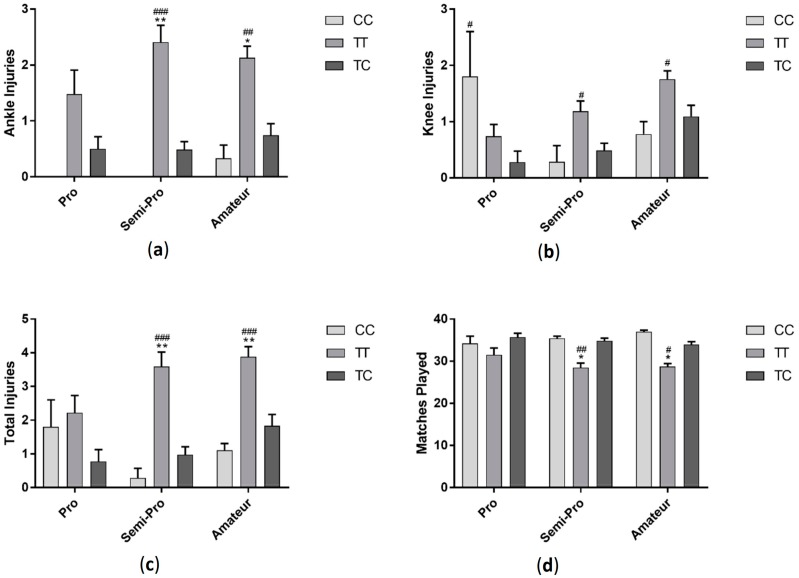
Effect of *GDF5* (rs143383) genotype on ankle injuries (**a**); knee injuries (**b**); total injuries (**c**) and matches played in professional, semi-professional and amateur soccer players (**d**). * *p* < 0.05, ** *p* < 0.01 compared to CC genotype, ^#^ *p* < 0.05, ^##^ *p* < 0.01, ^###^ *p* < 0.001 compared to TC genotype. Data shown as Mean ± S.E.M.

Players with TT genotype had significantly more ankle injuries (*F*_(2, 286)_ = 31.31 *p* < 0.001) and total injuries (*F*_(2, 286)_ = 30.69 *p* < 0.001) compared to players with CC and CT genotypes and had more knee injuries (*F*_(2, 286)_ = 11.06 *p* < 0.001) compared to those in the CT group (Figure 1). Those with TT genotype also played significantly less matches than players with CC and CT genotypes (*F*_(2, 287)_ = 21.35 *p* < 0.001). When data was sorted by profession, semi-professional and amateur players followed a similar trend, with those with TT genotype having more ankle (semi-professional: *F*_(2, 94)_ = 18.2 *p* < 0.001, amateur: *F*_(2, 143)_ = 11.05 *p* < 0.001), knee (semi-professional: *F*_(2, 94)_ = 5.5 *p* < 0.01, amateur: *F*_(2, 143)_ = 4.74 *p* < 0.05) and total injuries (semi-professional: *F*_(2, 94)_ = 16.12 *p* < 0.001, amateur: *F*_(2, 143)_ =11.85 *p* < 0.001) and playing significantly fewer matches (semi-professional: *F*_(2, 94)_ = 14.26 *p* < 0.001, amateur: *F*_(2, 143)_ = 15.01 *p* < 0.001). There was no difference in injury (ankle injuries: *F*_(2, 43)_ = 2.89 *p* > 0.05, knee injuries: *F*_(2, 43)_ = 4.25 *p* < 0.05, total injuries: *F*_(2, 43)_ = 2.47 *p* > 0.05) or matches played (*F*_(2, 43)_ = 2.33 *p* > 0.05) between genotypes in professional players (Figure 2).

### 3.2. AMPD1 Genotype Effect on Injury and Matches Played

**Figure 3:**
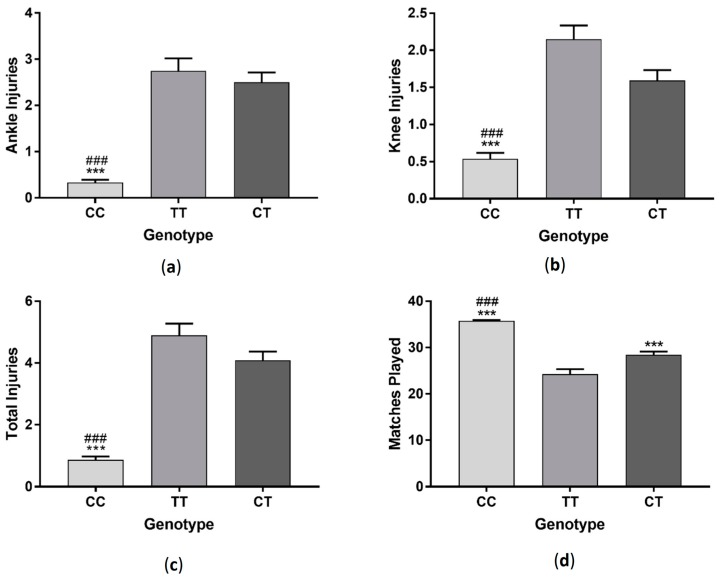
Effect of *AMPD1* (rs17602729) genotype on ankle injuries (**a**); knee injuries (**b**); total injuries (**c**) and matches played in a group of soccer players (**d**). *** *p* < 0.001 compared to TT genotype, ^###^ *p* < 0.001 compared to CT genotype. Data shown as Mean ± S.E.M.

**Figure 4:**
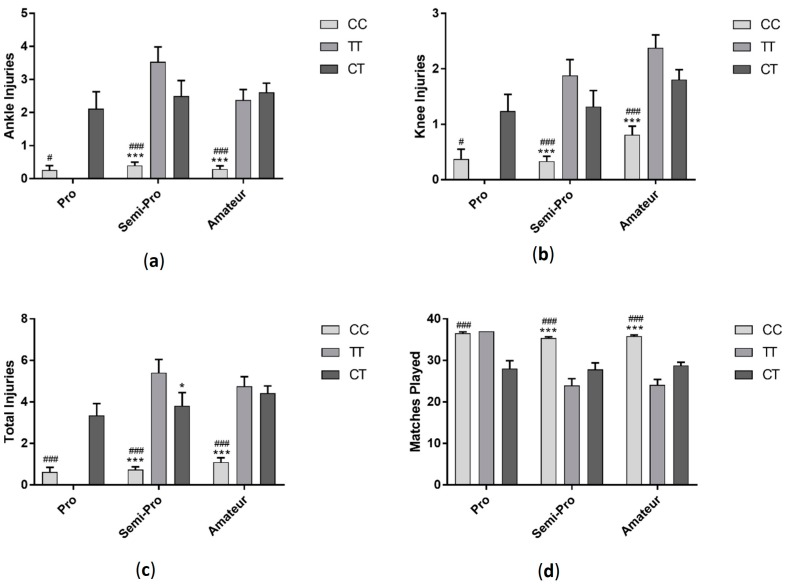
Effect of AMPD1 (rs17602729) genotype on ankle injuries (**a**); knee injuries (**b**); total injuries (**c**) and matches played in professional, semi-professional and amateur soccer players (**d**). *** *p* < 0.001 compared to TT genotype. ^#^ *p* < 0.05, ^###^ *p* < 0.001 compared to CT genotype. Data shown as Mean ± S.E.M.

Soccer players with CC genotype had significantly fewer ankle (*F*_(2, 282)_ = 77.85 *p* < 0.001), knee (*F*_(2, 282)_ = 44.04 *p* < 0.001) and total injuries (*F*_(2, 282)_ = 101.40 *p* < 0.001) compared to players with TT and CT genotypes. Those in the CC group also played significantly more matches (*F*_(2, 282)_ = 78.66 *p* < 0.001) than TT and CT groups (Figure 3). When data was sorted by profession, each profession category followed a similar pattern, with professional, semi-professional and amateur players with CC genotype having significantly fewer ankle (professional: *F*_(2, 42)_ = 9.14 *p* < 0.01, semi-professional: *F*_(2, 93)_ = 39.73 *p* < 0.001, amateur: *F*_(2, 141)_ = 34.46 *p* < 0.001), knee (professional: *F*_(2, 42)_ = 3.68 *p* < 0.05, semi-professional: *F*_(2, 93)_ = 20.81 *p* < 0.001, amateur: *F*_(2, 141)_ = 17.06 *p* < 0.001) and total injuries (professional: *F*_(2, 42)_ = 13.77 *p* < 0.001, semi-professional: *F*_(2, 93)_ = 46.77 *p* < 0.001, amateur: *F*_(2, 141)_ = 40.8 *p* < 0.001) and playing significantly more matches (professional: *F*_(2, 42)_ = 15.54 *p* < 0.001, semi-professional: *F*_(2, 93)_ = 44.45 *p* < 0.001, amateur: *F*_(2, 141)_ = 57.06 *p* < 0.001) than players with TT and CT genotypes (Figure 4).

### 3.3. COL5A1 Genotype Effect on Injury and Matches Played

**Figure 5:**
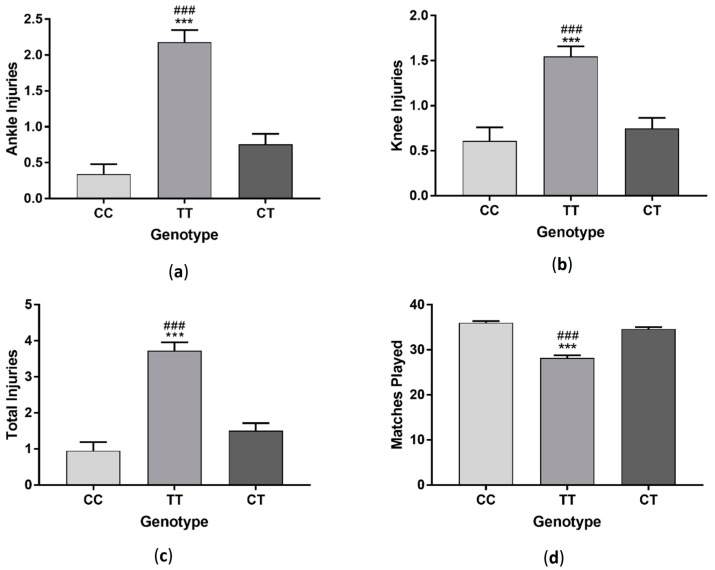
Effect of *COL5A1* (rs12722) genotype on ankle injuries (**a**); knee injuries (**b**); total injuries (**c**) and matches played in a group of soccer players (**d**). *** *p* < 0.001 compared to CC genotype, ^###^ *p* < 0.001 compared to CT genotype. Data shown as Mean ± S.E.M.

**Figure 6:**
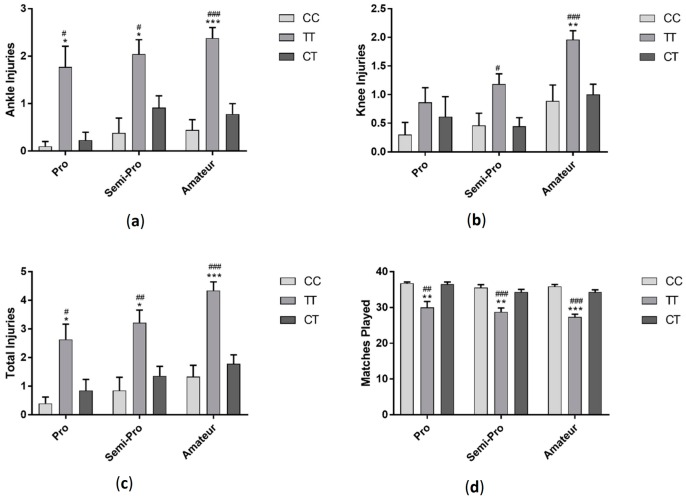
Effect of *COL5A1* (rs12722) genotype on ankle injuries (**a**); knee injuries (**b**); total injuries (**c**) and matches played in professional, semi-professional and amateur soccer players (**d**). * *p* < 0.05, ** *p* < 0.01, *** *p* < 0.001 compared to CC genotype. ^#^ *p* < 0.05, ^##^ *p* < 0.01, ^###^ *p* < 0.001 compared to CT genotype. Data shown as Mean ± S.E.M.

Soccer players with TT genotype had significantly fewer ankle (*F*_(2, 287)_ = 29.95 *p* < 0.001), knee (*F*_(2, 287)_ = 16.18 *p* < 0.001) and total injuries (*F*_(2, 287)_ =34.78 *p* < 0.001) compared to players with CC and CT genotypes (Figure 5). Those in the TT group also played significantly more matches (*F*_(2, 287)_ = 33.28 *p* < 0.001) than CC and CT groups (Figure 3). When data was sorted by profession, each profession category followed a similar pattern, with professional, semi-professional and amateur players with TT genotype having significantly fewer ankle (professional: *F*_(2, 42)_ = 6.51 *p* < 0.01, semi-professional: *F*_(2, 95)_ = 6.43 *p* < 0.01, amateur: *F*_(2, 144)_ = 17.47 *p* < 0.001), knee (professional: *F*_(2, 42)_ =0.87 *p* > 0.05, semi-professional: *F*_(2, 95)_ =5.65 *p* < 0.01, amateur: *F*_(2, 144)_ = 10.28 *p* < 0.001) and total injuries (professional: *F*_(2, 42)_ = 5.99 *p* < 0.01, semi-professional: *F*_(2, 95)_ = 7.75 *p* < 0.01, amateur: *F*_(2, 144)_ = 21.83 *p* < 0.001) and playing significantly more matches (professional: *F*_(2, 42)_ = 7.83 *p* < 0.01, semi-professional: *F*_(2, 95)_ = 11.52 *p* < 0.001, amateur: *F*_(2, 144)_ = 30.66 *p* < 0.001) than players with CC and CT genotypes (Figure 6).

### 3.4. IGF2 Genotype Effect on Injury and Matches Played

**Figure 7:**
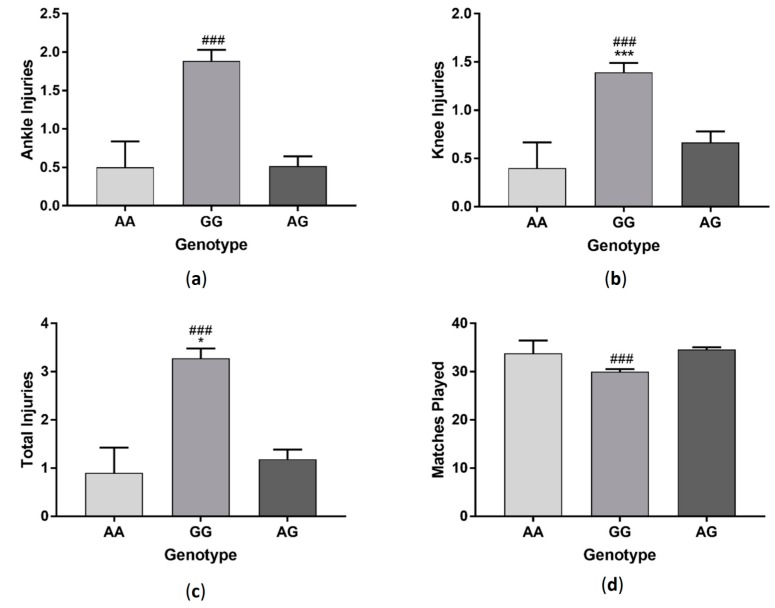
Effect of *IGF2* (rs680) genotype on ankle injuries (**a**); knee injuries (**b**); total injuries (**c**) and matches played in a group of soccer players (**d**). *** *p* < 0.001 compared to AA genotype, ^###^ *p* < 0.001 compared to AG genotype. Data shown as Mean ± S.E.M.

**Figure 8:**
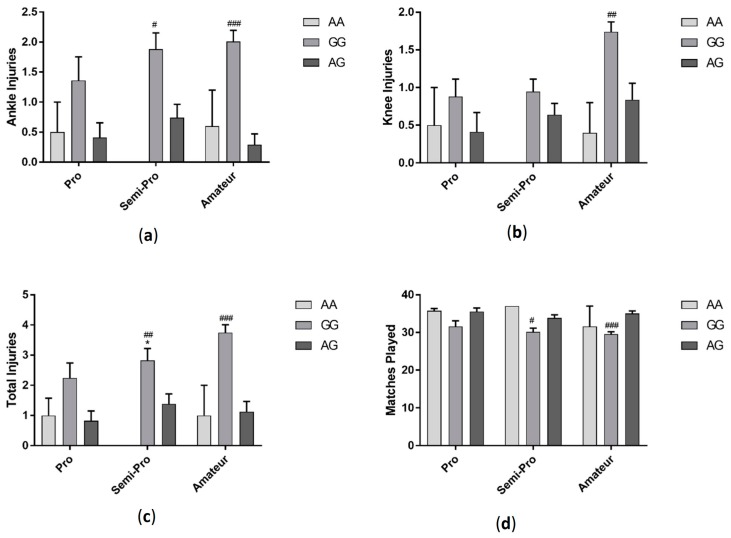
Effect of *IGF2* (rs680) genotype on ankle injuries (**a**); knee injuries (**b**); total injuries (**c**) and matches played in professional, semi-professional and amateur soccer players (**d**). * *p* < 0.05 compared to AA genotype, ^#^ *p* < 0.05 ^##^ *p* < 0.01, ^###^ *p* < 0.001 compared to AG genotype. Data shown as Mean ± S.E.M.

Players with GG genotype had significantly more knee (*F*_(2, 288)_ = 11.37 *p* < 0.001) and total injuries (*F*_(2, 288)_ = 22.58 *p* < 0.001) compared to players with AA and AG genotypes (Figure 7). Those with GG genotype also had significantly more ankle injuries (*F*_(2, 288)_ = 19.24 *p* < 0.001) and played fewer matches (*F*_(2, 288)_ = 16.10 *p* < 0.001) compared to those with AG genotype. Amateur players with GG genotype had significantly more ankle, knee and total injuries and played fewer matches (ankle injuries: *F*_(2, 144)_ =11.89 *p* < 0.001, knee injuries: *F*_(2, 144)_ = 7.1 *p* < 0.01, total injuries: *F*_(2, 144)_ = 14.32 *p* < 0.001, matches played: *F*_(2, 144)_ = 8.62 *p* < 0.001). Semi-professional players had more knee and total injuries and played fewer matches, but there were no significant differences between genotypes in the number of ankle injuries sustained (ankle injuries: *F*_(2, 95)_ = 4.77 *p* < 0.05, knee injuries: *F*_(2, 95)_ = 1.12 *p* > 0.05, total injuries: *F*_(2, 95)_ = 3.83 *p* < 0.05, matches played: *F*_(2, 95)_ = 4.08 *p* < 0.05). Although there was a similar trend, there were no significant differences between genotypes in ankle, knee or total injuries or in the number of matches played amongst professional players (ankle *F*_(2, 43)_ = 1.89 *p* > 0.05, knee *F*_(2, 43)_ =0.94 *p* > 0.05, total *F*_(2, 43)_ = 2.58 *p* > 0.05, matches *F*_(2, 43)_ = 2.44 *p* > 0.05) (Figure 8).

### 3.5. Correlation between Matches Played and Injuries

**Figure 9:**
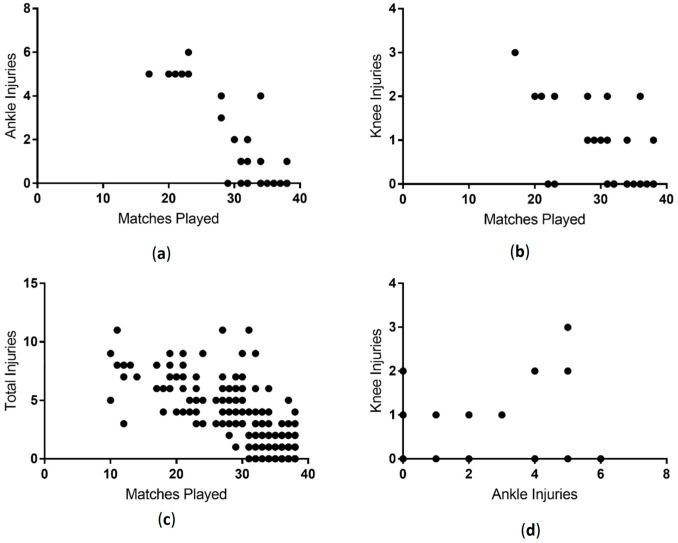
Correlation between injuries and matches played. An increase in ankle (**a**); knee (**b**) and total injuries (**c**) negatively correlated with the number of matches played. An increase in knee injuries positively correlated with an increase in ankle injuries (**d**).

Given that recovery from knee and ankle injuries in soccer players often leads to missed match time, we sought to determine if there was a correlation between the numbers of injuries players had and the number of matches played. Indeed, we found that ankle, knee and total injuries were significantly negatively correlated with matches played (ankle r = −0.74, *p* < 0.001, knee r = −0.57, *p* < 0.001, total injuries r = −0.79, *p* < 0.001) (Figure 9). Interestingly we also found that knee and ankle injuries were significantly positively correlated (r = 0.43, *p* < 0.001), suggesting that having a knee injury makes a player more likely to also sustain an ankle injury, and vice versa.

## 4. Discussion

Injury is becoming an increasing problem among soccer players. JLT Speciality [10] reported that injuries cost premier league clubs £79M during the first half of the 2016/17 season. It is also estimated that every year teams lose an average of 15% of working days to injury [11]. Not to mention the cost that losing star players for key matches would have on the performance and ranking of a team. Given these high costs, even a small reduction in the number of injuries could reduce expenses enormously. However, in order to reduce injury rates, the underlying risk factors for injury must first be elucidated.

Genes are now becoming increasingly recognised as key players in athletic performance and as risk factors for sports injury. Therefore, this study aimed to identify genes associated with ankle and knee injuries in professional, semi-professional and amateur soccer players. Moreover, we also sought to determine if these genes had an impact on the number of matches played in a season. The study focused on 4 genes that are thought to play some role in muscle pain, fatigue and damage. These included, *GDF5*, *AMPD1*, *COL5A1* and *IGF2*.

We found that players expressing the TT genotype of the *GDF5* gene had an increased number of ankle, knee and total injuries and a decrease in the number of matches played. When the data was sorted by profession, there was a similar trend in semi-professional and amateur players. This aligns with previous findings that showed that the T allele of *GDF5* was associated with ACL rupture [12] and Achilles tendinopathy [13]. This is likely due to reduced levels of the GDF5 protein in players carrying TT genotype leading to disruption of ligament homeostasis [14]. Interestingly, there were no differences in injuries or number of matches played between genotypes in professional players. Given that professional players are under a lot of physical strain at the top level, it is possible that even expressing a normal amount of *GDF5* is not sufficient to protect a player from sustaining and injury.

We found that players with the CC genotype of the *AMPD1* gene had significantly fewer ankle, knee and total injuries and played significantly more matches than those expressing a T allele. This was also true for professional, semi-professional and amateur players. Although research on the association between *AMPD1* and injury is limited, we have previously shown that weightlifters with CT or TT genotypes experience more pain following training sessions and needed longer recovery times [15]. This suggests that soccer players with T variants might also require longer recovery between training sessions and matches, but are not given sufficient rest time and therefore have higher injury rates than players with the CC genotype.

In our study, players with the TT genotypes of the *COL5A1* gene had an increased number of injuries and a decreased number of matches played overall and in professional, semi-professional and amateur groups. Previous studies have shown that the CC genotype of *COL5A1* appears to be protective against Achilles tendinopathy [16,17] and to be associated with reduced incidence of ACL rupture [18]. Although, these findings somewhat align with our study, we have further narrowed down that the TT genotype seems to be particularly vulnerable to ankle and knee injuries even when compared to the CT genotype. Brown et al. [19] the increased injuries in this group in soccer players.

Players with the GG genotype of the *IGF2* gene had an increased number of injuries and reduction in the number of matches played overall. The same was true for semi-professional and amateur players. Although professional soccer players followed a similar trend with GG having a slight increase in injury and decrease in matches, this did not reach significance. Previously, Pruna et al. [20] showed that individuals with the GA genotype had fewer severe injuries than either GG or AA genotypes. We have also shown that the GG genotype is associated with more injuries than even the AA genotype.

Finally, we analysed the correlation between injuries and matches played. Given that recovery from knee and ankle injuries in soccer players often leads to missing matches, we sought to determine if higher injury resulted in less matches played. As we expected, we found a significant negative correlation between ankle, knee and total injuries and matches played. This means that the more injuries a player had the less matches they played. This is important as the cost of losing a player for matches can be significant in terms of paying for treatment and salary and the cost to rankings in a league. Interestingly, we also found that knee and ankle injuries were also highly correlated, suggesting that sustaining one type of injury predisposes a player to a second type.

It is important to note that although some genotypes are more susceptible to injury than others, genetic testing should not be used to as an indicator of athletic performance and should not discount a potentially valuable player who is predisposed to injury. Genetic testing should be used to identify players who are prone to injury and adapt their training accordingly. In fact some of the genotypes that we have found to be associated with injury have also been shown to be associated with elite performance. Brown et al. [19] reported better endurance running performance in patients with TT genotype of *COL5A1* compared to participants with TC or CC and there is a high prevalence of the GG genotype of the *IGF2* gene amongst top level sprinters and jumpers [21].

## 5. Overall Limitations and Conclusions

The limitations of the study can be linked to the participant numbers in certain categories; data linked with professional players is of higher quality, as more information is recorded on them when compared with semi-pro or amateur players. The study therefore had to forsake information on the professional players to allow for a homogenous look at higher numbers of participants. Future study could gain more information on specific mechanisms of injury and link these to the genes identified in this study.

A second limitation is that epigenetic change is not taken into account; this is due to the high cost and difficulty in getting samples. Epigenetic change may or may not impact injury levels; however, base DNA analysis as done in this study is required before epigenetic testing can be accurately carried out, and therefore future studies may wish to build upon this.

Overall, it is clear that certain genes are linked with injury occurrence regardless of which level of soccer is played. Common sense and the study shows that injuries are directly correlated to matches missed, which is highly costly for sports teams. The gene *AMPD1* has been shown to be linked with a need for increased recovery after hard activity, and those who do take this rest actually perform better [15]. By looking at the genes analysed here, it would be possible to create a cost-effective one-time test that may help coaching staff personalise training and nutrition to help keep players injury-free.

By creating a small gene panel test, it would be possible to analyse players once in their lifetime and help staff conclude on rehab methods (aggressive vs normal), rest periods for players, and training intensities, and it could be used in a battery of other tests to help decide on player fitness.

## Supplementary Materials

The following are available online at http://www.mdpi.com/2075-4663/6/1/21/s1.
